# Ganymede Observations by JunoCam on Juno Perijove 34

**DOI:** 10.1029/2022GL099211

**Published:** 2022-12-12

**Authors:** M. A. Ravine, C. J. Hansen, G. C. Collins, P. M. Schenk, M. A. Caplinger, L. Lipkaman Vittling, D. J. Krysak, R. P. Zimdar, J. B. Garvin, S. J. Bolton

**Affiliations:** ^1^ Malin Space Science Systems San Diego CA USA; ^2^ Planetary Science Institute Tucson AZ USA; ^3^ Wheaton College Norton MA USA; ^4^ Lunar and Planetary Science Institute/USRA Houston TX USA; ^5^ NASA Goddard Space Flight Center Greenbelt MD USA; ^6^ Southwest Research Institute San Antonio TX USA

**Keywords:** Ganymede, Juno, JunoCam, geology, topography

## Abstract

During the Juno Mission's encounter with Ganymede on 7 June 2021, the Juno camera (JunoCam) acquired four images of Ganymede in color. These images covered one‐sixth of Ganymede at scales from 840 m to ∼4 km/pixel. Most of this area was only previously imaged by Voyager 1 in 1979, at lower spatial resolution and poorer image quality. No changes were observed over this area of Ganymede in the 42 years since Voyager. JunoCam provided overlapping coverage, from which we developed a digital elevation model of the best‐resolved area. A 3 km high dome at the subjovian point was confirmed, 450 km by 750 km. We used the JunoCam images to refine the geologic map of Ganymede in eastern Perrine Regio.

## Key Points

- In 2021, Juno camera (JunoCam) acquired close‐up images of Ganymede, providing improved spatial resolution relative to the 1979 Voyager 1 coverage
- While no changes were observed in the 42 years since Voyager, previously unrecognized paterae and craters were identified
- Topography derived from JunoCam confirmed a 3 km high dome at Ganymede's subjovian point and showed it to be elliptical in planform

## 1 Introduction

**Figure 1:**
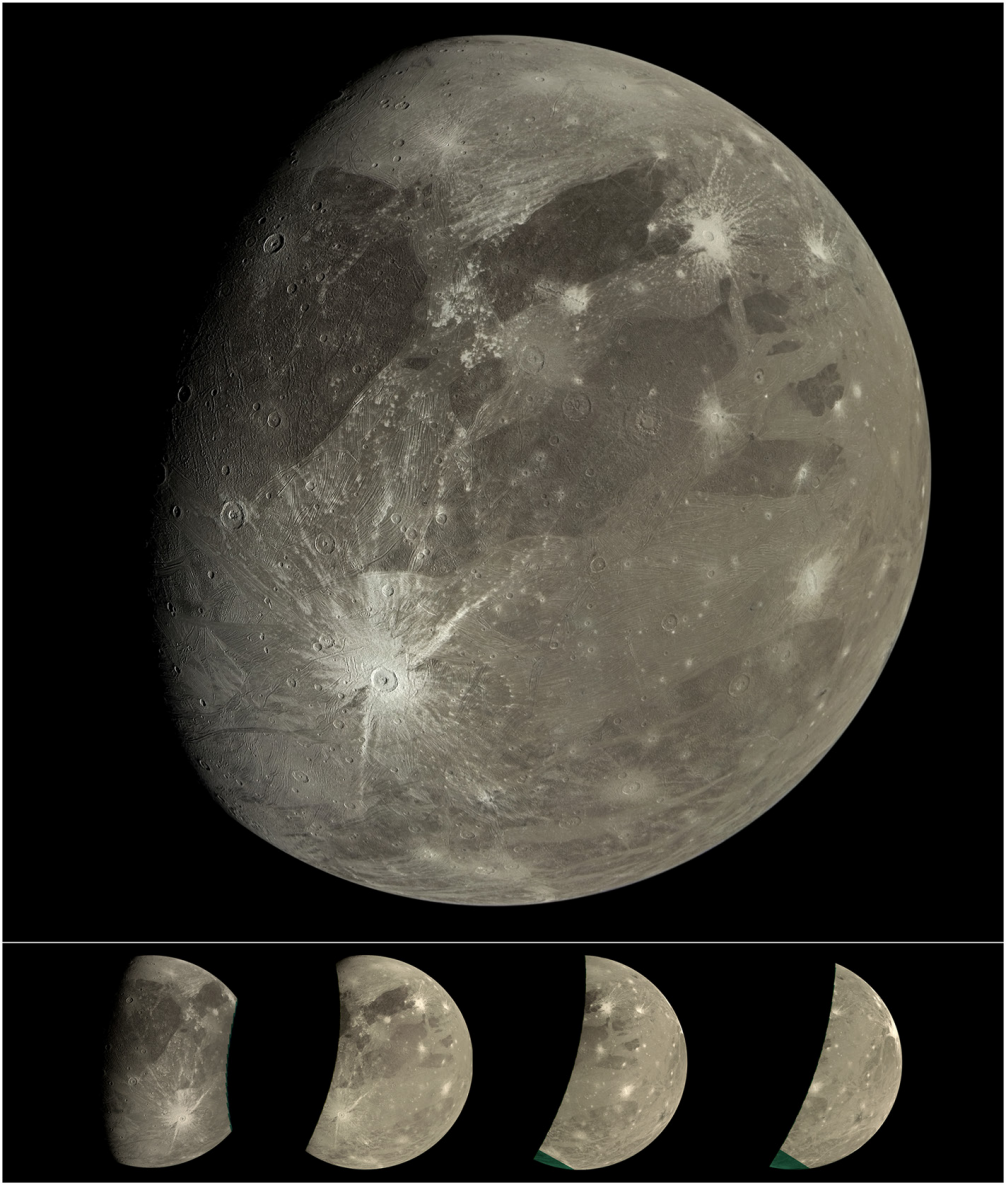
Upper: a mosaic of the first two perijove 34 (PJ34) Juno camera (JunoCam) images of Ganymede (north up, illumination from the right). Crater Tros, southwest of the center of the image, is 92 km in diameter. Lower: the four JunoCam images acquired on PJ34. The elongate crater near the center of images 3 and 4, Nanshe Catena, is 100 by 50 km. Images were acquired from 2021 to 06‐07T16:57:20 to 2021‐06‐07T17:00:22. NASA/JPL‐Caltech/SwRI/MSSS/Björn Jónsson.

The Juno Jupiter orbiter's camera, Juno camera (JunoCam), acquires wide field of view color images. It was designed to image Jupiter's polar regions from Juno's highly elliptical orbit (Hansen et al., 2017). Late in Juno's primary mission, there was a close encounter with Ganymede. The extended mission will have close flybys of Europa (2022) and of Io (2023 and 2024). Here we report initial results from the Ganymede encounter on perijove 34 (PJ34). A mosaic of two JunoCam images of the PJ34 Ganymede sequence is shown in the upper image in Figure 1.

## 2 PJ34 Ganymede Images

Juno flew by Ganymede west to east, with a minimum altitude of 1,046 km. After crossing the terminator, JunoCam acquired four images in color (red‐green‐blue), separated by 60 s (Figure 1, lower). Spacecraft rotation scans 70 RGB JunoCam “framelets” for each image. The altitude above Ganymede increased from 1,256 km (first image) to 3,372 km (fourth), increasing the nadir scale from 0.84 km/pixel to 2.27 km/pixel. The incidence angles range from 90° (first image) to 0° (fourth).

These four images cover from south of 10°S to north of 60°N and from ∼40°W to ∼40°E, including eastern Perrine Regio and Sicyon Sulcus in the north and Phrygia Sulcus and Barnard Regio to the south. The first two images provide particularly good coverage of the 92 km diameter, youthful crater, Tros. The subjovian point is in the southern part of the second and third images (Figure 1).

Further details of Juno's Ganymede encounter are included in Hansen et al. (2022).

## 3 Methods

### 3.1 Comparison of JunoCam With Voyager 1 Coverage

**Figure 2:**
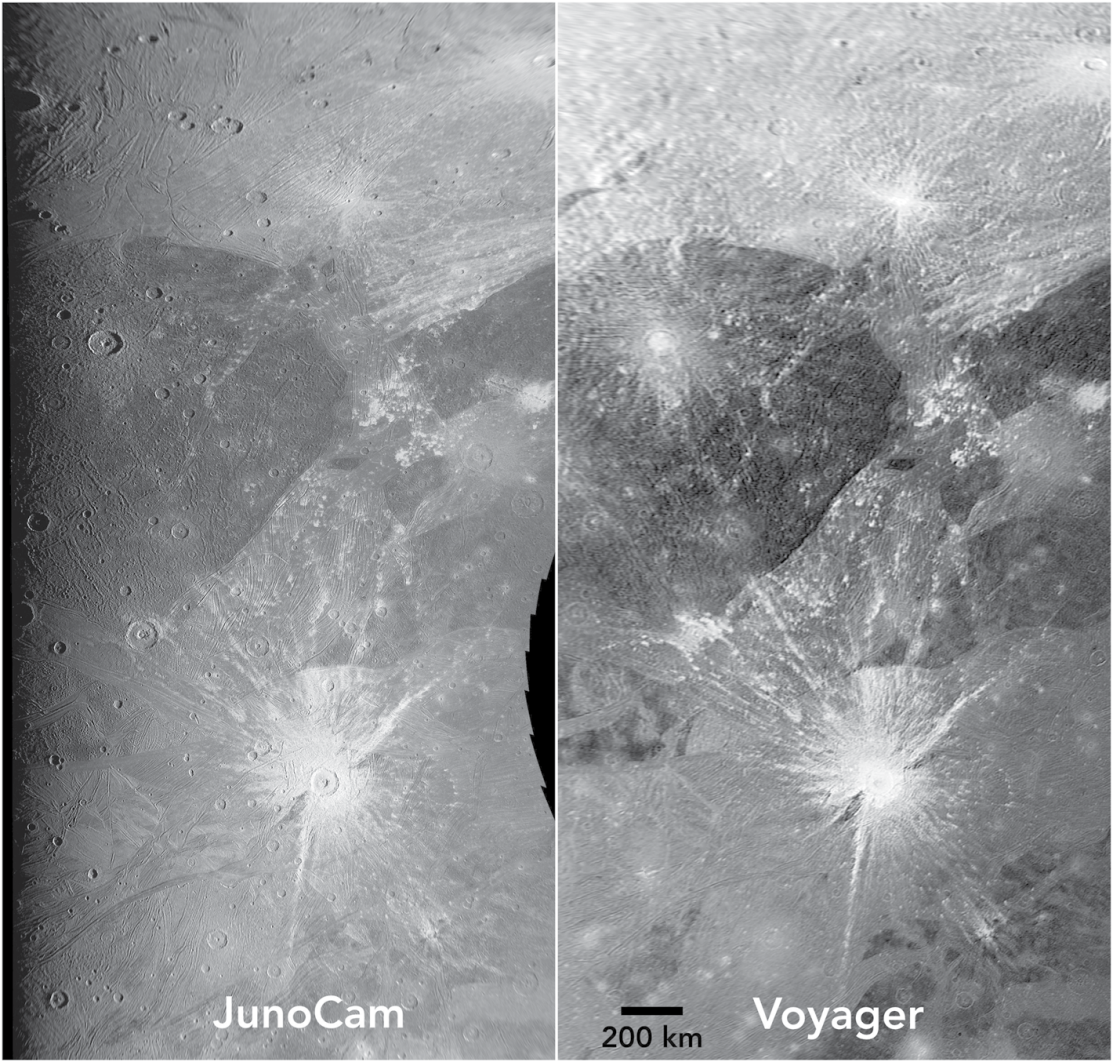
Left: The Juno camera (JunoCam) image 1 of the Phrygia/Tros region. Illumination is from the right, north at the top. Right: a mosaic of the same area from the best Voyager images, 1 km/pixel. The incidence angle of the Voyager images is less than 20°. NASA/JPL‐Caltech/SwRI/MSSS.

To compare the JunoCam PJ34 images with previous Voyager coverage of Ganymede, we reprojected both data sets into mosaics in a common simple cylindrical projection at a scale of 1 km per pixel. This sampling is a reasonable representation of the intrinsic scale of the JunoCam images, and matches the scale of the Voyager 1 Imaging Sub‐system (ISS) and Galileo Solid State Imager (SSI) Ganymede coverage (Becker et al., 2001; P. M. Schenk, 2010). Some Galileo SSI coverage was at a smaller pixel scale, but covered <20% of JunoCam's image area, and was not used for the comparison. Voyager 1 images of JunoCam's imaging region vary from 1.5 to 2.5 km/pixel and emission angles from 30° to 70°. Figure 2 compares the first JunoCam image with the Voyager mosaic at the same projection and scale. The JunoCam images provide better discriminability having been acquired at a higher incidence angle with higher resolution. Also, JunoCam has a higher modulation transfer function (MTF) and greater within‐scene dynamic range than Voyager 1 ISS (11‐bits vs. 8‐bits). Discriminability becomes progressively poorer in JunoCam images 3 and 4, as distance to Ganymede increased and incidence angle decreased.

To determine whether new impact features formed in this region since 1979, the two registered, map projected mosaics were compared by flickering. Ratio images of the JunoCam and Voyager mosaics were also used to search for changes but interpretations were complicated by the strong changes in brightness in low‐ and high‐Sun areas of the two mosaics. More sophisticated methods of comparison will likely be required once higher resolution global remapping of Ganymede is acquired in the 2030s.

### 3.2 Topographic Mapping

Stereogrammetry methods (see P. M. Schenk et al., 2022) were applied to the JunoCam images to derive topography over most of the observed area. Spacecraft motion resulted in sufficient parallax between images to generate digital elevation models (DEMs) with vertical precisions of several hundred meters. This is sufficient to resolve the deeper craters but not features such as grooved bright terrains or furrows, which have vertical relief less than several hundred meters (P. M. Schenk et al., 2022; Squyres, 1981). Pixel‐scale shape‐from‐shading topographic mapping techniques (P. M. Schenk et al., 2022) were also applied to the terminator regions to derive local scale relief.

Separately, we derived a DEM using all of the JunoCam framelets covering Tros using a tailored structure‐from‐motion (SfM) algorithm (Garvin et al., 2017, 2018, 2019), constrained vertically by shadow length estimates of the Tros central pit depth. This independent method agreed with the standard stereogrammetric approach to within ∼10%, with variances at high spatial frequencies. This level of agreement provides confidence that JunoCam‐based DEM's at new spatial scales (i.e., ground scale ≤1 km) can be computed for targets such as Ganymede.

## 4 Results

### 4.1 Changes Since 1979

A fundamental question for the satellites in the Jovian system concerns the ages of geologic terrains and the dynamical events (such as tidal heating episodes) connected to their formation. While new geologic features are unlikely due to the great age of the surface (Zahnle et al., 1998), new impact features are possible given ongoing impact events into Jupiter's atmosphere. Age dating of terrains from impact crater density relies on constraints on impact fluxes in the system (e.g., Zahnle et al., 2003). The 42‐year gap between Voyager (1979) and JunoCam (2021) provides an opportunity to search for new impact features in this region of Ganymede and potentially constrain the current impactor flux over a finite time, since the Galileo imaging coverage in this region was too sparse to provide a useful constraint.

A flicker comparison between the registered JunoCam and Voyager reprojected mosaics (see Methods) revealed no apparent new impact features. Given the high albedo of fresh craters on Ganymede, with high albedo ejecta deposits two or three times the diameter of the craters themselves, we argue that new craters as small as 250 m diameter would be detectable in images at these 1 km per pixel scales. Extrapolating Ganymede cratering rates from Zahnle et al. (2003) below 1 km, the probability of JunoCam observing a new crater over 12.2 million km^2^ in 42 years is 1 in 1500, consistent with none being observed.

### 4.2 Topographic Analysis

**Figure 3:**
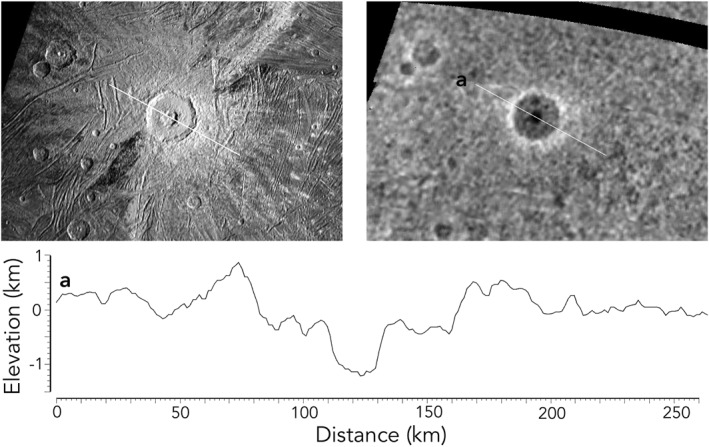
Upper left: Crater Tros, imaged by Juno camera (JunoCam). Upper right: digital elevation model (DEM) derived from JunoCam stereo coverage. Bottom: a cross‐section of the DEM across Tros along line “a,” showing a rim to floor depth of ∼1 km and a central pit depth of ∼750 m.

**Figure 4:**
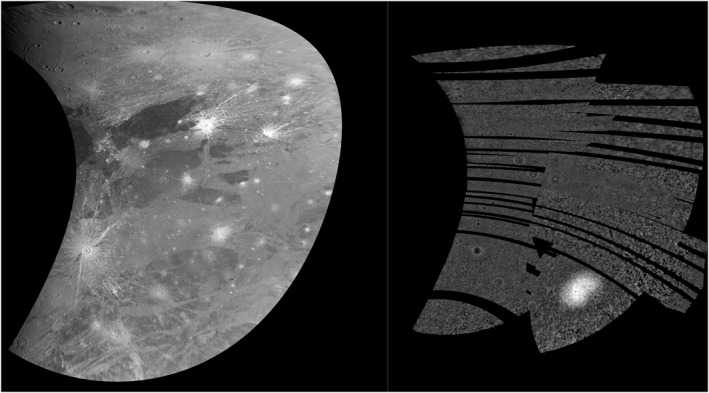
Left: Cylindrical projection of Juno camera (JunoCam) images 2 and 3, Tros near the western edge, Perrine Regio in the north and Barnard Regio to the south. Right: the JunoCam digital elevation model using the same projection. While the topography of the larger craters is resolved, the amplitude of that topography (∼1 km) is modest compared with the 750 km by 450 km dome (∼3 km), overlapping the subjovian point.

Digital elevation maps (DEMs; Figures 3 and 4) derived from JunoCam stereo images (see Methods) are not sufficient to resolve geologic structures but do resolve roughly 10 craters >30 km across in the mapped area, including the 92‐km central pit crater Tros. Tros has one of the most extensive bright ray systems and is, therefore, one of the youngest large craters on Ganymede. Tros was poorly resolved by Voyager due to the high incidence angle. The JunoCam mosaic at ∼1 km/pixel and our new DEM reveals a raised rim and a broad, flat floor with a 750‐m‐deep central pit and a small residual central dome offset from crater center (Figure 3). The depth of ∼1 km is consistent with prior measured depths of well‐preserved complex craters on Ganymede (P. Schenk, 2002). While most craters this size have larger central domes, the smaller size of Tros's dome is consistent with the variation in crater morphology on Ganymede (Moore & Malin, 1988; P. M. Schenk, 2010; P. M. Schenk et al., 2004).

Determining the age of Tros is relevant to age determinations and flux rates in the Jovian system. No younger superposed craters >5 km are evident on the floor or proximal ejecta of Tros (abundant secondary craters are visible ∼1 crater diameter from the rim, however). Within these limits, this indicates a maximum age of impact between <1.6 and 3.6 Ga, assuming the flux rates from Zahnle et al. (2003). The lower limit presumes no craters >5 km within one‐half the Tros radius of the rim; the upper limit presumes no craters >5 km only over the Tros floor. An improved estimate of Tros age will be possible with higher resolution from future missions.

The most prominent feature in the JunoCam DEM is the 3‐km‐high dome (Figure 4) centered at 1.5°E, 0.5°N (the subjovian point) originally discovered by P. M. Schenk et al. (2022) in limb scans and partial Voyager stereo coverage. The dome does not correlate with known geologic structures or terrain boundaries, and unlike the putative dome at Zakar crater (Squyres, 1980) is not correlated with any impact structure (P. M. Schenk et al., 2022), or with the much smaller central domes of central dome craters on Ganymede and Callisto (Moore & Malin, 1988; P. M. Schenk et al., 2004). This dome could be the product of localized tidal heating or migration of a polar dome (formed by ice shell thickening due to lower temperatures [Ojakangas & Stevenson, 1989]) to the equator owing to polar wander (P. M. Schenk et al., 2022).

The JunoCam DEM fully maps this dome as a ∼450 by 700 km oval. The DEM shows no other topography of near this amplitude in the northern subjovian hemisphere, highlighting the dome's uniqueness. Small‐scale geologic features related to possible dome growth or collapse, such as radial/circumferential fractures or ridges, are not evident in JunoCam or prior imaging.

### 4.3 Geological Observations and Interpretations

**Figure 5:**
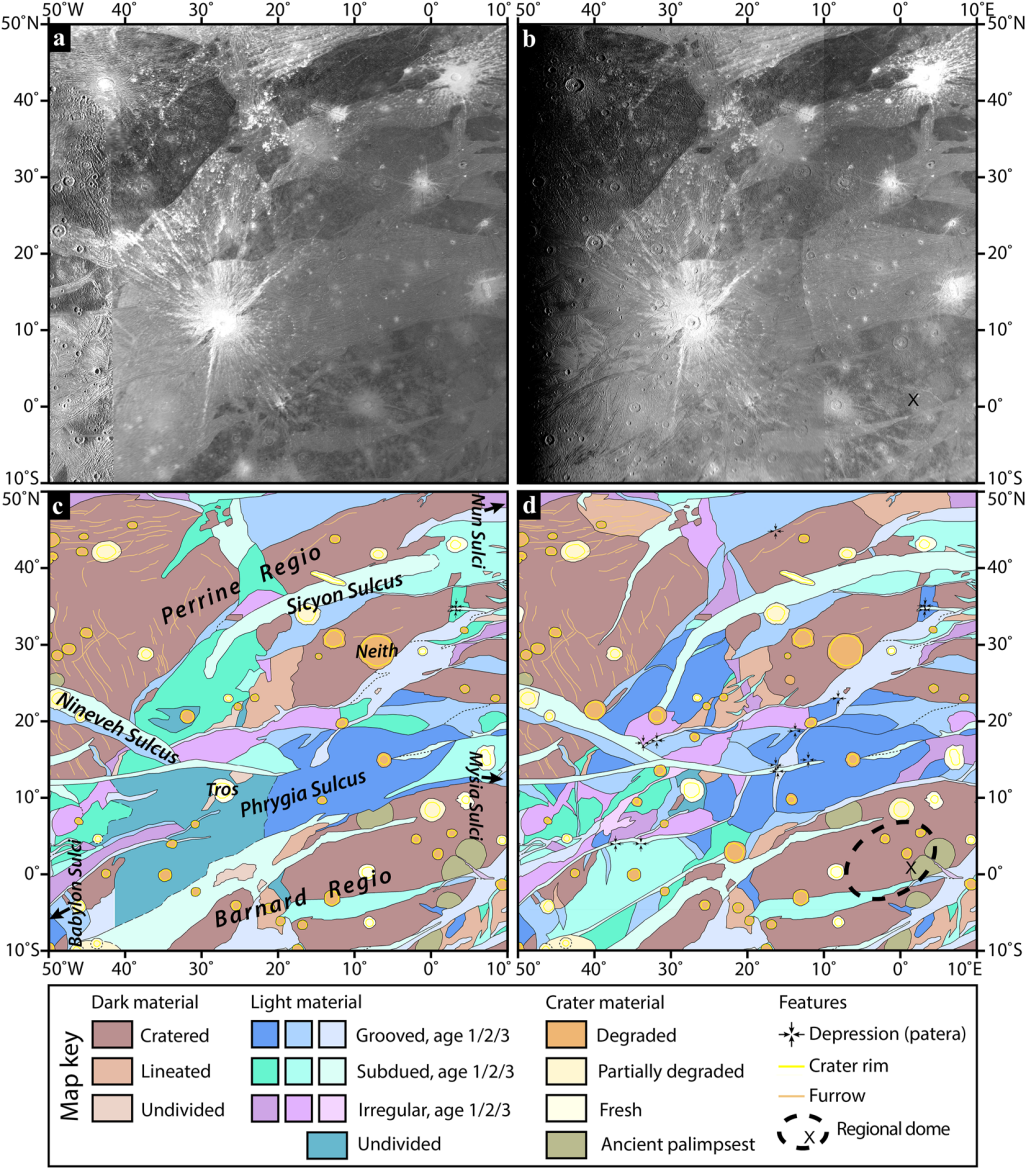
(a) Mosaic of Voyager/Galileo coverage of Ganymede in simple cylindrical projection, covering the area discussed in the text (credit: USGS); (b) First JunoCam image of the same area; (c) Excerpt from the global geologic map of Ganymede (Collins et al., 2013). The simplified key uses the same unit definitions as the USGS map (Patterson et al. (2010); Collins et al., 2013), ages 1/2/3 in light material refer to relative ages based on cross‐cutting relationships from age 1 (oldest) to age 3 (youngest); (d) Preliminary reinterpretation of geologic map based on JunoCam data, using the same unit definitions in panel (c) The regional‐scale topographic dome in Section 4.2 is shown by the dashed outline, subjovian point indicated by “X.”.

JunoCam images covering Phrygia Sulcus, Sicyon Sulcus, and eastern Perrine Regio present a major improvement over Voyager 1 coverage for the purposes of geological mapping (Section 2). In the previous Voyager 1 coverage, low resolution, moderate to high emission angle, low solar incidence angle, and high albedo rays from Tros crater served to mask details of high‐albedo terrain features in this region. Comparison between the new and old maps in Figure 5 shows several differences (Sections 4.4.3 and 4.4.4), including terrain boundaries previously mapped as “undivided” or as “approximate” (Collins et al., 2013; see Figure 5c), several large craters and 12 paterae newly identified in this region (Sections 4.4.1 and 4.4.2).

#### 4.3.1 Crater Material

The Collins et al. (2013) geologic map only included impact craters >30 km diameter identified with high confidence. The new map shows five additional degraded impact craters in this size range. Two of these craters (101 km diameter at 21.6°N, 40.1°W, and 47 km at 25.5°N, 14.6°W) were identified from Voyager by P. M. Schenk et al. (2004) but not included in the 2013 map owing to low confidence. The other three craters (110 km diameter at 3°N, 21.7°W; 52 km at 13.6°N, 31.5°W; 41 km at 18°N, 27°W) were not previously reported, but are faintly visible in Voyager 1 images. One feature mapped as a degraded crater in the 2013 map was revealed by JunoCam to be a patera (see next section). A 34 km trough, oriented NW‐SE at 15.5°N, 324°E, appears to be an impact feature, not a tectonic feature, and may be a catena. There are also two possible craters >30 km near the southwestern end of Sicyon Sulcus, but they appear highly relaxed and tectonically modified.

#### 4.3.2 Paterae

**Figure 6:**
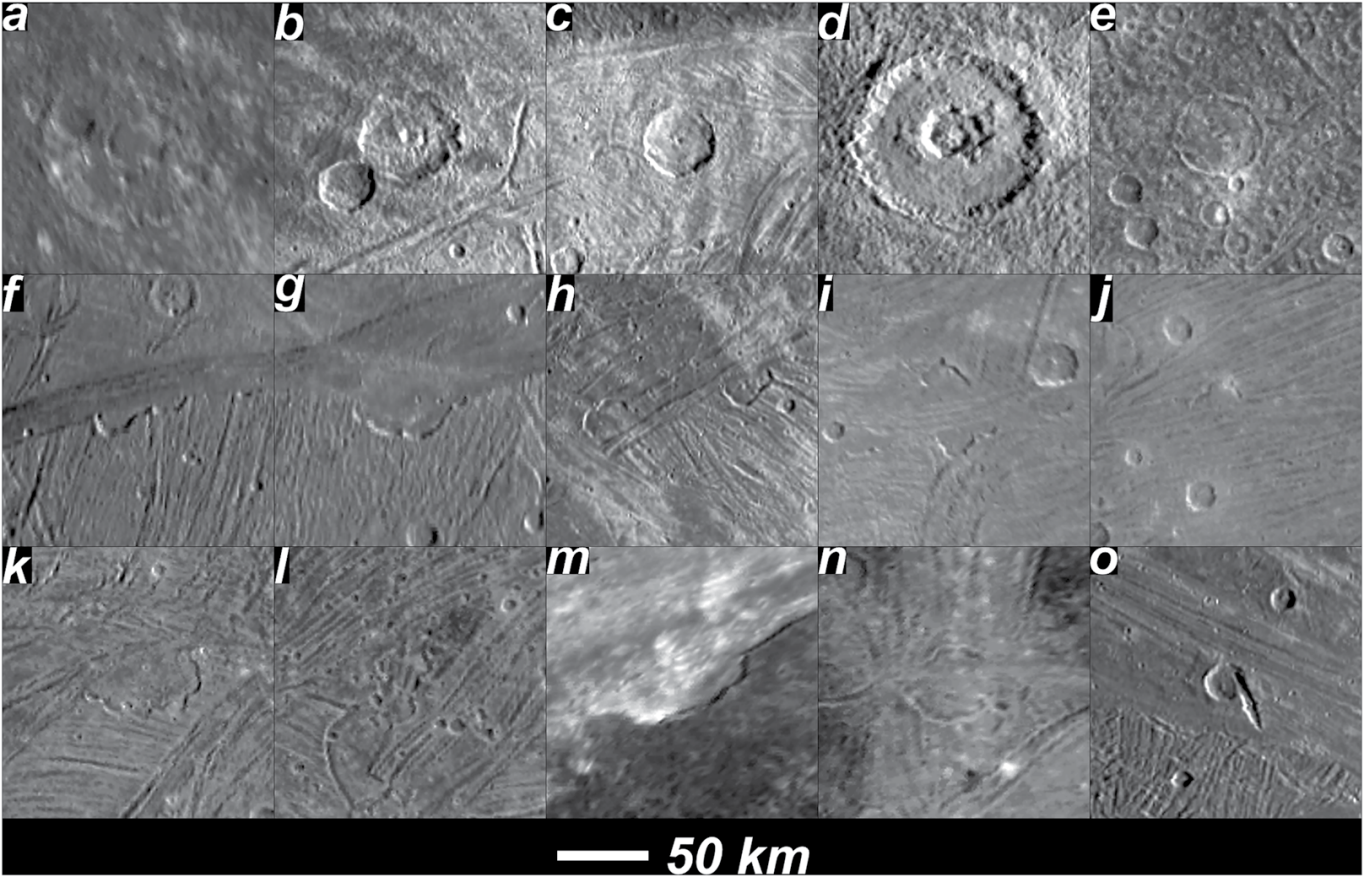
Upper row, first three images from left, three craters identified by Juno camera (JunoCam) not previously identified (*a*: 110 km diameter at 3°N, 21.7°W; (b) 52 km at 13.6°N, 31.5°W; (c) 41 km at 18°N, 27°W); fourth and fifth images are craters previously seen in Voyager data but not mapped (d): 101 km diameter at 21.6°N, 40.1°W; (e) 47 km at 25.5°N, 14.6°W). Lower two rows, 12 paterae were observed by JunoCam, 10 not previously identified by Voyager (the newly identified 10 are (f): 4.0°N, 37.2°W; (g): 3.9°N, 33.9°W; (h) (2): 17.1°N, 33.6°W and 17.5°N, 31.9°W; (i) (2): 13.8°N, 16.3°W; (j) 15.0°N, 12.1°W; (k) 18.7°N, 13.8°W; (l): 23.1°N, 8.0°W; (m): 44.8°N, 16.7°W. The previously identified paterae are in (n): 35.2 N, 3.3°E and 34.6°N 3.2°E. Image (o) shows a possible previously unrecognized catena: 15.5°N, 36°W. Flicker animations between the JunoCam and Voyager coverage are included as Supporting Information S1.

Caldera‐like depressions with scalloped edges, known as paterae, were previously observed on Ganymede. P. Schenk and Moore (1995) mapped 18 of them in Voyager data. Paterae may be linked to possibly gas‐rich (cryo)volcanic processes involved in the formation of light material on Ganymede (Allison & Clifford, 1987; P. M. Schenk et al., 2001). JunoCam imaging revealed 12 paterae, only two of which are evident in the high‐sun Voyager data. Like paterae elsewhere on Ganymede, they are almost all adjacent to and truncated by the most recent light (usually subdued) material units, indicating they are formed by late‐stage volcanic processes. The 12 paterae seen in this region (shown in Figures 5d and 6) now represent about a quarter of 47 possible paterae on Ganymede, an atypical concentration. The only other region on Ganymede with such a concentration is in Mummu Sulci identified in the extensive Voyager 2 anti‐Jovian hemisphere coverage (Head et al., 1999; P. Schenk and Moore, 1995), curiously almost antipodal to the Phrygia/Sicyon region. Whether such an antipodal arrangement relates to tidal heating patterns or other internal dynamics, or whether there are concentrations of paterae hiding in other poorly imaged areas of Ganymede, are interesting topics for investigation once global mapping by the JUICE mission begins in the 2030s.

#### 4.3.3 Light Material

Using the JunoCam images, the undivided light terrain around Tros (Collins et al., 2013) can be subdivided into a network of crosscutting grooves and smooth areas. Networks of young (age 3), interconnected, grooved and subdued light terrain units are found to the east (Mysia Sulci and Nun Sulci) and west (Babylon Sulci) of this area, and the new map shows these adjacent structures are connected from east to west. In particular, the young, subdued terrain in Nineveh Sulcus can now be seen to clearly link with the young subdued terrain that forms the southern margin of Perrine Regio. These structures link with young grooved units along the northern margin of Phrygia Sulcus, which continue into Nun Sulci. The young, subdued and grooved units along the southern boundary of Phrygia are now distinguished from the surrounding, older light material units, which run into the network of young grooves along the northern part of Mysia Sulci, off the eastern edge of Figure 5.

An area mapped as lineated dark material near 25°N, 20°W can now be confidently subdivided into dark material and narrow lanes of light material. The pattern of dark terrain breakup here is reminiscent of patterns of recent grooves seen in Mysia Sulci off the eastern edge of the map, and patterns in the area where Dardanus Sulcus breaks up Barnard Regio, off the southern edge of the map.

#### 4.3.4 Dark Material

Minor revisions were made to dark material unit boundaries, providing confidence that dark material was correctly mapped in 2013. Undivided dark material can now be subdivided into cratered and lineated dark material. Two substantial areas in northern Perrine Regio that were mapped as dark cratered material (Figure 5c) are now seen a better match to dark lineated material (Figure 5d).

Two sets of furrows were observed in Perrine Regio in Voyager and Galileo images (oriented NNW‐SSE and WSW‐ ENE). JunoCam fails to clearly show the WSW‐ ENE furrows. While this may be an effect of differing illumination between the images, the JunoCam images call into question the true extent of this particular furrow set in Perrine Regio.

## 5 Conclusions

JunoCam imaged 12.2 million km^2^ of Ganymede's northern subjovian hemisphere, significantly improving the quality of available image coverage and topographic mapping in that region. No changes were detected at scale greater than 250–500 m over the area in the 42 years since it was first imaged by Voyager 1. JunoCam imaged 10 paterae and five large craters not previously mapped in high‐Sun Voyager images, serving as a cautionary example to the planetary science community about drawing conclusions from a single set of images. The higher quality JunoCam coverage has been used to significantly improve the geologic map over this area. Stereoscopic observation of topography from the JunoCam stereo images reveal prominent topographic features, including Tros crater and the large topographic dome at the subjovian point. The insight gained from this handful of images makes it likely in our opinion that new observations from the upcoming JUICE and Europa Clipper missions will revolutionize our understanding of Ganymede.

## Supporting information

Supporting Information S1

Movie S1

Movie S2

Movie S3

Movie S4

Movie S5

Movie S6

Movie S7

Movie S8

## Data Availability

This research made use of JunoCam images archived with the PDS (Caplinger, 2014) and the USGS Integrated Software for Imagers and Spectrometers (ISIS), https://doi.org/10.5281/zenodo.3962369.
