# Supplementary material for: Ganymede Observations by JunoCam on Juno Perijove 34
Source: Geophys Res Lett. 2022 Dec 12;49(23):e2022GL099211. doi: 10.1029/2022GL099211 (PMC10078141; doi:10.1029/2022GL099211)
Supplement: Supplementary file 1 — Supporting Information S1 [file GRL-49-0-s009.pdf]

AGU PUBLICATIONS

*Geophysical Research Letters*

Supporting Information for

# Ganymede Observations by JunoCam on Juno Perijove 34

**M.A. Ravine[1], C.J. Hansen[2], G.C. Collins[3], P.M. Schenk[4], M.A. Caplinger[1], L. Lipkaman Vittling[2], D. J. Krysak[1], R. P. Zimdar[1], J.B. Garvin[5] and S.J. Bolton[5]**

[1]Malin Space Science Systems

[2]Planetary Science Institute

[3]Wheaton College, Massachusetts

[4]Lunar and Planetary Science Institute/USRA

[5]NASA Goddard Space Flight Center

[6]Southwest Research Institute

Corresponding author: Michael Ravine (ravine@msss.com)

## Contents of this file

Captions for Data Set ds1 through ds8

## Additional Supporting Information (Files uploaded separately)

ds1_fig6a_crater_blink.gif
ds2_fig6b_crater_blink.gif
ds3_fig6c_crater_blink.gif
ds4_fig6d_crater_blink.gif
ds5_fig6fg_patera_blink.gif
ds6_fig6h_patera_blink.gif
ds7_fig6i_patera_blink.gif
ds8_fig6k_patera_blink.gif

## Introduction

These supplementary materials consist of animations of eight of the areas on Ganymede covered in Figure 6 (craters or paterae), that flicker between the JunoCam images and

the Voyager coverage of the same areas, provided to illustrate the differences in surface discriminability between the JunoCam and Voyager coverage.

- These animations are in Graphics Interchange Format (gif) format
- They switch back-and-forth between the JunoCam and Voyager images of the areas in question;
- Each of the JunoCam and Voyager images was map projected into the same geometry (simple cylindrical, as discussed in the paper).

The captions for the eight animation files are as follows:

**DS1.** ds1_fig6a_crater_blink.gif. A GIF animation of a large crater identified by JunoCam not previously identified (Shown in Figure 6a: 110 km diameter at 3°N, 21.7°W). The animation switches between the JunoCam view and the Voyager view, showing the better surface feature discriminability provided by the JunoCam coverage.

**DS2.** ds2_fig6b_crater_blink.gif. A GIF animation of a large crater identified by JunoCam not previously identified (Shown in Figure 6b: 52 km diameter at 13.6°N, 31.5°W). The animation switches between the JunoCam view and the Voyager view, showing the better surface feature discriminability provided by the JunoCam coverage.

**DS3.** ds3_fig6c_crater_blink.gif. A GIF animation of a large crater identified by JunoCam not previously identified (Shown in Figure 6c: 41 km diameter at 18°N, 27°W). The animation switches between the JunoCam view and the Voyager view, showing the better surface feature discriminability provided by the JunoCam coverage.

**DS4.** ds4_fig6d_crater_blink.gif. A GIF animation of a large crater identified by JunoCam not previously identified (Shown in Figure 6d: 101 km diameter at 21.6°N, 40.1° W). The animation switches between the JunoCam view and the Voyager view, showing the better surface feature discriminability provided by the JunoCam coverage.

**DS5.** ds5_fig6fg_patera_blink.gif. A GIF animation of two paterae identified by JunoCam not previously identified (Shown in Figure 6f: 4.0°N, 37.2°W and Figure 6g: 3.9°N, 33.9°W). The animation switches between the JunoCam view and the Voyager view, showing the better surface feature discriminability provided by the JunoCam coverage.

**DS6.** ds6_fig6h_patera_blink.gif. A GIF animation of two paterae identified by JunoCam not previously identified (Shown in Figure 6h (2): 17.1°N, 33.6°W and 17.5°N, 31.9°W). The animation switches between the JunoCam view and the Voyager view, showing the better surface feature discriminability provided by the JunoCam coverage.

**DS7.** ds7_fig6i_patera_blink.gif. A GIF animation of two paterae identified by JunoCam not previously identified (Shown in Figure 6i (2): 13.8°N, 16.3°W). The animation

switches between the JunoCam view and the Voyager view, showing the better surface feature discriminability provided by the JunoCam coverage.

**DS8.** ds8_fig6k_patera_blink.gif. A GIF animation of a paterae identified by JunoCam not previously identified (Shown in Figure 6k: 18.7°N, 13.8°W). The animation switches between the JunoCam view and the Voyager view, showing the better surface feature discriminability provided by the JunoCam coverage.
