# Supplementary figures and images for: Ganymede Observations by JunoCam on Juno Perijove 34
Source: Geophys Res Lett. 2022 Dec 12;49(23):e2022GL099211. doi: 10.1029/2022GL099211 (PMC10078141; doi:10.1029/2022GL099211)

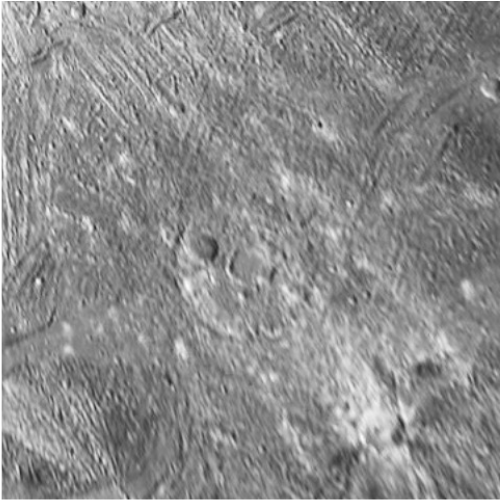

Supplement: Supplementary file 2 — Movie S1 [file GRL-49-0-s005.gif]

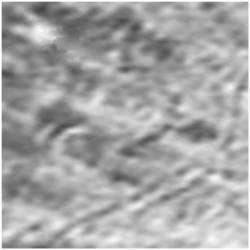

Supplement: Supplementary file 3 — Movie S2 [file GRL-49-0-s001.gif]

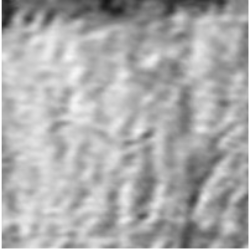

Supplement: Supplementary file 4 — Movie S3 [file GRL-49-0-s003.gif]

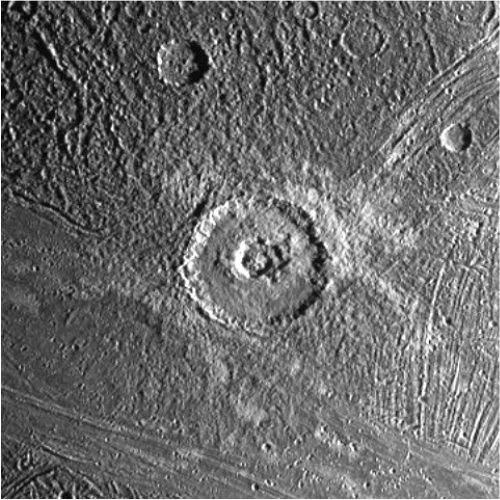

Supplement: Supplementary file 5 — Movie S4 [file GRL-49-0-s006.gif]

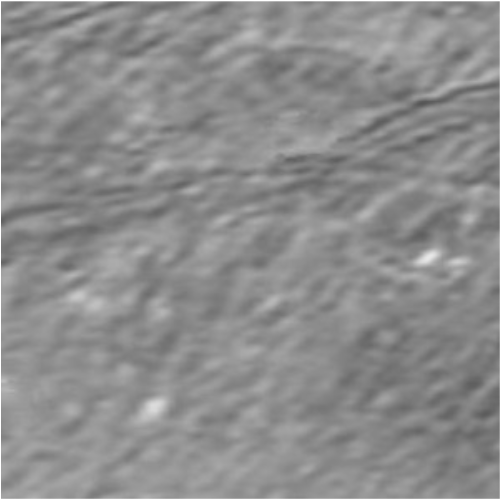

Supplement: Supplementary file 6 — Movie S5 [file GRL-49-0-s007.gif]

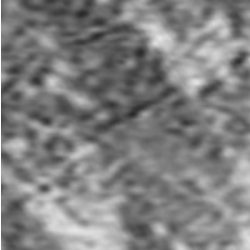

Supplement: Supplementary file 7 — Movie S6 [file GRL-49-0-s002.gif]

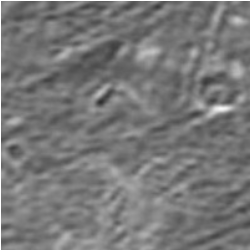

Supplement: Supplementary file 8 — Movie S7 [file GRL-49-0-s008.gif]

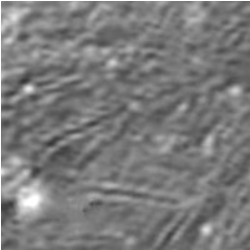

Supplement: Supplementary file 9 — Movie S8 [file GRL-49-0-s004.gif]
